# Excessive Pretreatment Weight Loss Is a Risk Factor for the Survival Outcome of Esophageal Carcinoma Patients Undergoing Radical Surgery and Postoperative Adjuvant Chemotherapy

**DOI:** 10.1155/2018/6075207

**Published:** 2018-01-28

**Authors:** Xiao-Li Yu, Jin Yang, Ting Chen, Yi-min Liu, Wei-ping Xue, Ming-Hui Wang, Shou-Min Bai

**Affiliations:** ^1^Guangdong Provincial Key Laboratory of Malignant Tumor Epigenetics and Gene Regulation, Department of Radiation Oncology, Sun Yat-sen Memorial Hospital, Sun Yat-sen University, Guangzhou, China; ^2^Department of Radiation Oncology, The Fifth Affiliated Hospital of Guangzhou Medical University, Guangzhou, China; ^3^Guangdong Provincial Key Laboratory of Malignant Tumor Epigenetics and Gene Regulation, Department of Cardiothoracic Surgery, Sun Yat-sen Memorial Hospital, Sun Yat-sen University, Guangzhou, China

## Abstract

**Background:**

The prognostic values of weight loss and body mass index (BMI) in esophageal carcinoma remain controversial. This study aimed to evaluate the impacts of weight loss on the survival of patients undergoing radical surgery and adjuvant chemotherapy.

**Methods:**

The medical records of 189 consecutive patients with nonmetastatic esophageal carcinoma treated in our hospital between January 2012 and December 2013 were reviewed, and 121 patients were included for analysis.

**Results:**

Kaplan-Meier analysis revealed that the 3-year overall survival rate was significantly higher in the low pretreatment weight loss (pre-LWL) group than in the high pretreatment weight loss (pre-HWL) group (*P* < 0.001). In addition, the 3-year overall survival rate of normal weight group was higher than that of overweight and underweight groups (*P* = 0.007). Multivariate Cox proportional hazards analysis showed that pre-LWL group had a significantly better 3-year overall survival than pre-HWL group (*P* = 0.027, HR = 1.89, and 95% CI = 1.07–3.32). pN stage and age were also the survival prognostic factors.

**Conclusions:**

Our study showed that low pretreatment weight loss predicted a better survival outcome in the esophageal carcinoma patients with radical surgery and adjuvant chemotherapy. However, BMI and weight loss during treatment had no impact on the survival outcome.

## 1. Introduction

Esophageal carcinoma is a highly aggressive neoplasm and is the eighth most common cancer worldwide [1]. The most patients are diagnosed at a locally advanced stage, with a long-term survival rate of around 10%–60% [2–4]. In 2008, there is an estimate of 482,000 patients newly diagnosed with esophageal carcinoma and 407,000 died of this disease [5]. Identification of prognostic factors of esophageal cancer is of clinical significance since it may be helpful to conduct clinical interventions to improve the prognosis of the high-risk patients. Currently, transthoracic esophagectomy with radical lymphadenectomy followed by postoperative adjuvant chemotherapy or radiotherapy is the standard curative treatment for locally advanced esophageal cancer [6].

Highly malignant tumors usually grow rapidly and metabolize actively and even lead to progressive body consumption and weight loss at diagnosis. This poor physical condition will hinder the treatment process, thereby affecting the survival. Excessive pretreatment weight loss has been shown to have an adverse effect on the survival outcome in many cancers, such as pancreatic cancer [7] and head and neck cancer [8]. Due to the tumor-related dysphagia, vomiting, reduced oral intake, and altered nutrient metabolism associated with systemic inflammation, weight loss is also a common presenting symptom of esophageal cancer [9–11]. On the other hand, both body weight and body mass index (BMI) are important factors for evaluating patient's nutritional status, tolerance to therapeutic interventions, and other considerations associated with the treatment response [12]. Hence, a number of studies have been conducted to investigate the effects of weight loss and BMI on the prognosis of esophageal cancer patients.

Until now, however, the impact of initial BMI on the survival of esophageal carcinoma patients remains contradictory [13–15]. Duan et al. have demonstrated that high BMI has a distinctly adverse impact on the long-term survival of esophageal squamous cell carcinoma patients after esophagectomy [14]. On the contrary, Miao et al. have reported that a high BMI is not associated with increased overall morbidity following esophagectomy [15]. Likewise, the influence of weight loss on the survival outcome of patients with esophageal cancer is also controversial [10, 16–18]. Van Der Schaaf et al. have revealed that a >10% preoperative weight loss leads to decrease of 5-year survival after esophageal cancer surgery [10], whereas Skipworth et al. have concluded that preoperative weight loss does not influence the perioperative mortality rate and short-term prognosis [18]. These findings suggest that the prognostic values of BMI and weight loss on esophageal cancer remain inconclusive and require further elucidation. Therefore, the purpose of this study was to evaluate the effects of weight loss and BMI on the survival outcomes of patients with esophageal carcinoma undergoing radical surgery and adjuvant chemotherapy.

## 2. Materials and Methods

### 2.1. Patients

The medical records of 189 consecutive nonmetastatic esophageal carcinoma patients treated in the Sun Yat-sen Memorial Hospital, Sun Yat-sen University, between January 2012 and December 2013, were reviewed. The diagnosis of esophageal carcinoma was made based on postoperative pathological examination. The inclusion criteria were as follows: (1) being newly diagnosed with squamous cell carcinoma; (2) being with nonmetastatic disease; (3) undergoing radical surgery followed by adjuvant chemotherapy. The exclusion criteria were as follows: (1) being without weight measurements at 6 months before diagnosis, at baseline, after surgery, and/or at the end of chemotherapy; (2) age > 70 years; (3) being without postoperative chemotherapy treatment; (4) being with any other cancers; (5) nonsquamous cell carcinoma. A total of 121 patients were included based on the criteria. This study was approved by the institutional review board (IRB) of Sun Yat-sen Memorial Hospital, Sun Yat-sen University, and written informed consent was waived by the IRB due to the retrospective nature of this study. All the data were analyzed anonymously.

### 2.2. Data Collection

Patient weight was recorded at the initial visit and weekly during hospitalization. Pretreatment weight loss was defined as unintentional weight loss during the 6 months before diagnosis. Preoperation weight was measured at the initial visit, and the final weight was recorded within 3 days before the final administration of chemotherapy. Weight loss during the whole treatment was defined as the difference between the preoperation weight and the final weight. Body mass index (BMI) was defined as the weight (kg) before the first chemotherapy divided by the square of height (meters) and was categorized according to the WHO recommendations for Asian populations [19, 20]. All patients were diagnosed by biopsy and restaged after radical surgery according to the International Union Against Cancer (UICC) 2009 Staging System [21, 22].

### 2.3. Surgery, Chemotherapy, and Follow-Up

All patients underwent transthoracic esophagectomy accompanied by mobilization of the stomach. The gastric tube was pulled up through the postmediastinal or retrosternal route, and a cervical or intrathoracic esophagogastric anastomosis was created. Total mediastinal and upper abdominal lymphadenectomy was routinely performed. Pathologic stage was determined according to the American Joint Committee on Cancer (AJCC) Staging System, 7th edition [21, 22].

Adjuvant chemotherapy was carried out at 2–6 weeks after operation, consisting of docetaxel and oxaliplatin for 3–6 cycles, 4 weeks/cycle. 20 patients received only 1-2 cycles.

After treatment completion, patients were followed up monthly in the first 3 months, then every 3 months during the first 3 years, and then every 6 months during the next 2 years. The duration of follow-up was calculated from the completion of treatment to the final visit or death. The final follow-up was on August 1, 2016.

### 2.4. Statistical Analysis

Data was analyzed using IBM SPSS Version 20 (SPSS Statistics V20, IBM Corporation, Somers, New York). The Chi-square test and Fisher's exact test (for expected value < 5) were used to compare ordinal and categorical variables. Due to the limited sample size, the pT stage was divided into T1-2 and T3-4; the pN stage was divided into N0, N1, and N2-3; the location of the primary lesion was divided into superior, middle, and inferior thoracic ones, and the cervical primary subgroup was combined with the superior thoracic subgroup. The overall survival rate (OS) was estimated using the Kaplan-Meier method and the differences in survival curves were compared by the log-rank test. Univariate and multivariate Cox proportional hazards models were used to evaluate the survival results of the weight loss or BMI-based subgroups with covariates including age, sex, pT stage, pN stage, the location of the tumor, and pathological classification. An independent variable with significance in both univariate and multivariate analyses would be recognized as an associated factor of survival outcome. Two-tailed *P* values < 0.05 were considered significant.

## 3. Results

### 3.1. Demographic and Clinical Characteristics of the Patients

A total of 121 patients with nonmetastatic esophageal carcinoma were included. The demographic and clinical characteristics were summarized in Table 1. The median duration of follow-up for all patients was 29 months (range: 1–55 months). For all patients, the range of pretreatment weight loss was −7.93% to 36.67% (median: 5.66%), and the range of weight loss during the whole treatment was −15.56% to 33.61% (median: 8.6%). Sixty-seven (55%) patients and 91 patients (75%) had a weight loss ≥ 5% before treatment and during treatment, respectively. Malnutrition is usually defined as a weight loss of more than 5% during the whole treatment [20, 23]. To investigate the effect of weight loss on the prognosis, patients were divided into dichotomous subgroups based on their rate of weight loss before treatment (high pretreatment weight loss [pre-HWL, weight loss ≥ 5%] versus low pretreatment weight loss [pre-LWL, weight loss < 5%] subgroups) and during treatment (HWL versus LWL).

The initial BMI of all the patients ranged from 14.84 kg/m^2^ to 29.43 kg/m^2^ (median: 21.51 kg/m^2^). The patients were divided into underweight (UW, BMI < 18.5 kg/m^2^, *n* = 14), normal weight (NW, BMI 18.5–24.99 kg/m^2^, *n* = 86), and overweight (OW, BMI ≥ 25 kg/m^2^, *n* = 21) groups based on their initial BMI. No patient had a BMI higher than 30 kg/m^2^.

There was no difference in gender, pN stage, and location of the primary lesion among the subgroups stratified by pretreatment weight loss, weight loss during the whole treatment, and BMI (*P* > 0.05, Table 1). The pre-HWL group had more patients with an age < 55 as compared with the pre-LWL group (*P* = 0.043, Table 1). The pre-LWL group, the HWL group, and the overweight group had more patients in early T stages (all *P* < 0.05, Table 1). The overweight group had more high and low differentiation in pathological classification while UW and NW group had more middle differentiation (*P* = 0.010, Table 1).

### 3.2. Univariate and Multivariate Analyses of 3-Year Overall Survival Stratified by the Weight Loss Status and BMI

**Figure 1:**
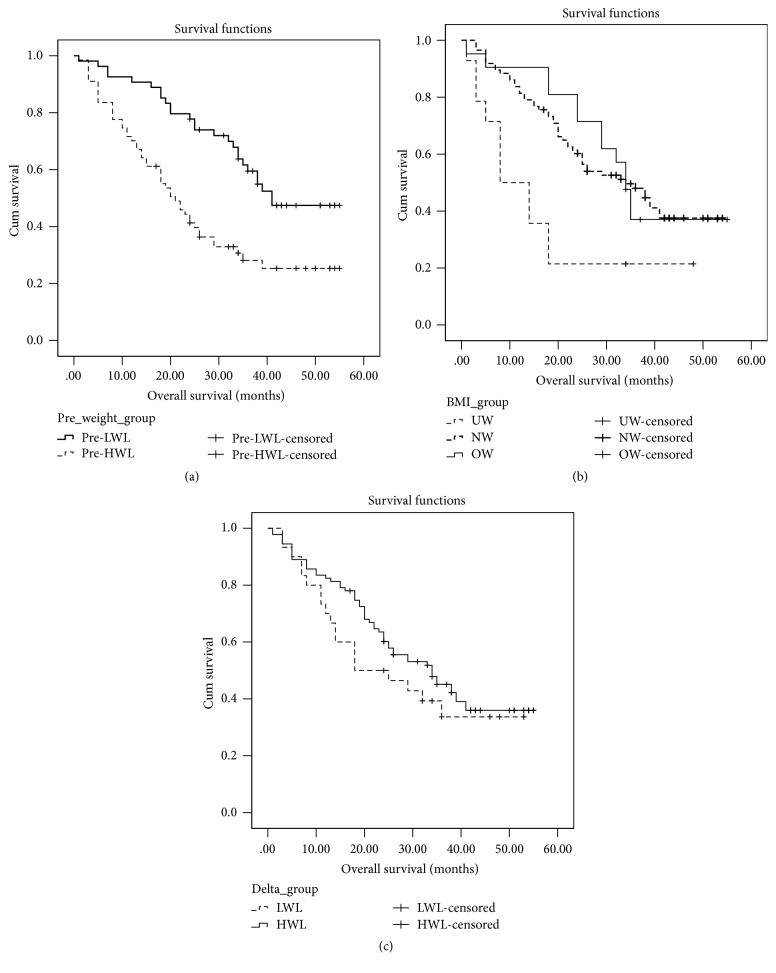
Kaplan-Meier survival curve analysis for the 3-year overall survival for the 121 patients with esophageal carcinoma undergoing surgery and adjuvant chemotherapy stratified by the (a) pretreatment weight loss (log-rank test, *P* < 0.001), (b) body mass index (*P* = 0.297), and (c) weight loss during the treatment (*P* = 0.007).

The 3-year overall survival rate for all patients was 39.7%. The 3-year overall survival rate was compared among the subgroups. Kaplan-Meier survival curve analysis revealed that pre-LWL group had a significantly better 3-year overall survival rate as compared with the pre-HWL group (51.9% versus 29.9%, log-rank test, *P* < 0.001; Table 2 and Figure 1(a)). In addition, normal weight group had significantly better 3-year overall survival rate than overweight and underweight groups (43.0% versus 38.1% versus 21.4%, log-rank test, *P* = 0.007; Table 2 and Figure 1(b)). However, the 3-year overall survival rate was not significantly different between the HWL and LWL groups (36.7% versus 40.7%, log-rank test, *P* = 0.297; Table 2 and Figure 1(c)).

The 3-year overall survival rate was also significantly different in the subgroups analyses stratified by the primary tumor location, pT stage, pN stage, age, and pathological classification (all *P* < 0.05, Table 2).

### 3.3. Independent Prognostic Factors of the 3-Year Overall Survival

To identify the independent prognostic factors of the 3-year overall survival, all the significant factors mentioned above were further investigated by univariate and multivariate Cox proportional hazards analysis. In univariate model, age, T stage, N stage, location, pretreatment weight loss, and BMI groups were the significant factors associated with 3-year overall survival (all *P* < 0.05, Table 3). In multivariate analysis, patients with low pretreatment weight loss had significantly better 3-year overall survival as compared with those with high pretreatment weight loss (*P* = 0.027, hazard ratio [HR] = 1.89, and 95% confidence interval [CI] = 1.07–3.32, Table 3). The other independent prognostic factors in the multivariable analysis were pN stage (N1: *P* = 0.017, HR = 2.14, and 95% CI = 1.14–3.99; N2: *P* = 0.014, HR = 2.31, and 95% CI = 1.18–4.51) and age (*P* = 0.015, HR = 0.51, and 95% CI = 0.29–0.87, Table 3).

## 4. Discussion

In this study, we investigated the effects of weight loss and BMI on the survival outcomes of patients with esophageal carcinoma receiving surgery and adjuvant chemotherapy. The results showed that the 3-year overall survival rate for all esophageal cancer patients was 39.7%. Kaplan-Meier analysis revealed that the 3-year overall survival rate was significantly higher in the pre-LWL group than in the pre-HWL group (*P* < 0.001). The 3-year overall survival rate of normal weight group was higher than that of overweight and underweight groups (*P* = 0.007). In addition, multivariate Cox proportional hazards analysis showed that pre-LWL group had a significantly better 3-year overall survival than pre-HWL group (*P* = 0.027, HR = 1.89, and 95% CI = 1.07–3.32). The pN stage and age (*P* < 0.05) were also identified as the independent prognostic factors for 3-year overall survival. Taken together, these results suggested that excessive pretreatment weight loss was a risk factor for the 3-year overall survival rate of esophageal carcinoma patients with surgery and adjuvant chemotherapy.

Our results showed that the pretreatment weight loss significantly affected the overall survival, which is consistent with Van Der Schaaf et al.'s study that revealed that an excessive preoperative weight loss (>10%) worsens the 5-year survival after esophageal cancer surgery [10]. In this study, we also observed that patients with a pretreatment weight loss ≥ 5% had a higher proportion of patients in the advanced T stages (T3-T4) as compared with those with pretreatment weight loss < 5% (*P* = 0.02), suggesting that patients with a high pretreatment weight loss were more likely to have an advanced tumor, thereby resulting in a poor survival outcome. In addition, a higher pretreatment weight loss may indicate a longer course of disease before diagnosis and less nutrient intake. Given that the pretreatment weight loss is not affected by nutritional intervention during treatment, it could more accurately reflect the status of disease progression. Our result may suggest that patients with a significant weight loss before treatment could be treated with early nutritional intervention to improve body weight, but whether it can prolong survival time remains to be further studied.

In this study, 75.2% (91/121) patients had weight loss ≥ 5% during the whole treatment, which was much higher than in Jiang et al.'s report. Their study showed that 40.3% of esophageal cancer patients had a weight loss ≥ 5% during radiotherapy [24]. This inconsistency may be attributed to the more severe complications (such as trauma, vomiting, and nutrition loss) induced by the combination of surgery and adjuvant chemotherapy as compared to those from radiotherapy alone. Our results showed that there was no difference in the 3-year overall survival between the two subgroups stratified by weight loss during the treatment. This result is consistent with a previous study [8] but conflicts with several studies that report that weight loss during treatment is an independent indicator of poor prognosis for esophageal carcinoma [3, 16, 17]. The different treatment modalities may contribute to this disagreement. The weight loss of patients in our study was more likely to be associated with the complex surgery, which caused a major trauma and a large energy consumption to the body, leading to a high weight loss and needed a long recovery time. However, weight loss in Cincibuch et al.'s [16] and Di Fiore et al.'s [3] studies is mostly caused by radiotherapy which leads to pharyngalgia, dysphagia, insufficient food intake, and esophagitis. These symptoms would be relieved and the weight may recover in a short time after the completion of radiotherapy. In addition, chemoradiotherapy itself is an independent prognostic factor of long-term survival [3]. Therefore, the impact of weight loss during treatment on the survival outcome may be different among patients receiving different treatments. Another possible explanation may be that weight loss during treatment may not accurately reflect the cumulative weight loss from the appearance of cancer to the completion of treatment since patients may experience a high weight loss before initial diagnosis but only a limited weight loss during the following treatment.

In this study, we found that BMI had no impact on the 3-year overall survival, which is in line with Miao et al.'s study that showed high BMI is not associated with increased overall morbidity following esophagectomy [15]. However, several studies have reported that high BMI is an independent prognostic factor of improved clinical outcome in cancer patients [20, 25, 26]. Thus, a large sample size study is necessary to further evaluate the impact of BMI on the survival outcome of esophageal cancer patients.

Our analysis showed that tumor N stage was an independent factor associated with 3-year overall survival. A lower N stage predicted a better overall survival, which is in agreement with the fact that lymph node metastasis predicts a poorer survival. On the other hand, our results also showed that patients with an age of ≥55 had a better 3-year overall survival rate. One of the possible explanations for this phenomenon is that young patients were at increased risk of developing more aggressive tumors as the trend found in breast cancer [27, 28], thus exhibiting a higher risk of cancer recurrence and death. Other factors analyzed in our multivariate analysis including sex, T stage, the location of the primary tumor or pathological type were not the independent factors for survival outcome.

There are still several limitations in our study. First, it is a retrospective study and the sample size was relatively small. In addition, there was no obese patient in our study. A well-designed prospective study with large sample size should be conducted to further validate the findings of this study. All these limitations should be addressed in the following study.

In summary, our study showed that, for patients with esophageal carcinoma who received radical surgery and adjuvant chemotherapy, a higher age, earlier pN stage, and lower pretreatment weight loss were the prognostic factors for better survival outcome. However, BMI and weight loss during treatment had no impact on survival outcome. Our findings are helpful for better understanding of the prognostic value of weight loss and BMI in the esophageal carcinoma patients with radical surgery and adjuvant chemotherapy.

## Figures and Tables

**Table 1:** Characteristics of the 121 patients with esophageal carcinoma enrolled in this study.

Characteristics	Pre-LWL	Pre-HWL	*P value* ^*∗*^	LWL	HWL	*P*	UW	NW	OW	*P value* ^*∗*^
Total	54	67		30	91		14	86	21	
Gender			0.732			0.432				0.407
Male	43	55		26	72		13	68	17	
Female	11	12		4	19		1	18	4	
Age (years)			0.043			0.082				0.353
<55	19	37		18	38		9	38	9	
≥55	35	30		12	53		5	48	12	
T stage^†^			0.020			<0.001				<0.001
T1-2	23	15		1	37		0	25	13	
T3-4	31	52		29	54		14	61	8	
N Stage^†^			0.231			0.369				0.201
N0	23	26		13	36		3	36	10	
N1	19	17		6	30		3	28	5	
N2-3	12	24		11	25		8	22	6	
Location			0.170			0.956				0.080
Cervical and superior	11	14		6	19		3	13	9	
Middle thoracic	37	37		18	56		7	57	10	
Inferior thoracic	6	16		6	16		4	16	2	
Pathology			0.577			0.839				0.010
High differentiation	17	17		8	26		3	21	10	
Middle differentiation	20	31		14	37		7	42	2	
Low differentiation	17	19		8	28		4	23	9	

Pre-LWL, low pretreatment weight loss; pre-HWL, high pretreatment weight loss; LWL, low weight loss during treatment; HWL, high weight loss during treatment; UW, underweight; NW, normal weight; OW, overweight; ^*∗*^*P* values were calculated using the *χ*^2^ test. ^†^According to the 7th AJCC/UICC staging system.

**Table 2:** Comparison of the 3-year overall survival in Kaplan-Meier analysis (*n* = 121).

	Overall survival (%)	*P* value^*∗*^
Weight loss		*P* = 0.297
LWL	36.7	
HWL	40.7	
Pretreatment weight loss		*P* < 0.001
Pre-LWL	51.9	
Pre-HWL	29.9	
BMI		*P* = 0.007
UW	21.4	
NW	43.0	
OW	38.1	
Location		0.025
Cervical and superior thoracic	16.0	
Middle thoracic	45.9	
Inferior thoracic	45.5	
N stage		<0.001
N0	63.3	
N1	25.0	
N2-3	22.2	
Age		0.021
<55	28.6	
≥55	49.2	
T stage		0.018
<55	55.3	
≥55	32.5	
Sex		0.678
Male	38.8	
Female	43.5	
Pathology		0.050
High differentiation	38.2	
Middle differentiation	51.0	
Low differentiation	25.0	

Pre-LWL, low pretreatment weight loss; pre-HWL, high pretreatment weight loss; LWL, low weight loss during treatment; HWL, high weight loss during treatment; UW, underweight; NW, normal weight; OW, overweight. ^*∗*^*P* values were calculated using the log-rank test.

**Table 3:** Summary of Cox-regression analyses of the prognostic factors for 3-year overall survival (*n* = 121).

Variables	Univariate		Multivariate	
	HR (95% CI)	*P*	HR (95% CI)	*P*
Gender		0.561		0.537
Male	Ref		Ref	
Female	0.84 (0.46–1.53)		0.82 (0.43–1.55)	
Age		0.006		0.015
<55	Ref		Ref	
≥55	0.52 (0.33–0.83)		0.51 (0.29–0.87)	
T stage		0.020		0.162
T1-2	Ref		Ref	
T3-4	1.91 (1.11–3.29)		1.56 (0.84–2.91)	
N stage		0.006		0.027
N0	Ref	-	Ref	-
N1	2.43 (1.33–4.41)	0.004	2.14 (1.14–3.99)	0.017
N2-3	2.37 (1.31–4.29)	0.004	2.31 (1.18–4.51)	0.014
Location		0.017		0.080
Cervical and superior thoracic	Ref	-	Ref	-
Middle thoracic	0.46 (0.27–0.79)	0.004	0.50 (0.27–0.92)	0.025
Inferior thoracic	0.54 (0.26–1.10)	0.091	0.58 (0.27–1.27)	0.175
Pathology		0.285		0.103
High differentiation	Ref	-	Ref	-
Middle differentiation	0.81 (0.45–1.46)	0.485	1.18 (0.61–2.29)	0.624
Low differentiation	1.26 (0.71–2.23)	0.425	1.89 (1.00–3.60)	0.051
Pretreatment weight loss		<0.001		0.027
Pre-LWL	Ref		Ref	
Pre-HWL	2.37 (1.46–3.86)		1.89 (1.07–3.32)	
Weight loss		0.305		0.497
LWL	Ref		Ref	
HWL	0.76 (0.45–1.28)		1.30 (0.61–2.75)	
BMI		0.011		0.545
UW	Ref	-	Ref	-
NW	0.38 (0.19–0.73)	0.004	0.61 (0.25–1.48)	0.275
OW	0.36 (0.16–0.80)	0.013	0.61 (0.22–1.73)	0.352

Pre-LWL, low pretreatment weight loss; pre-HWL, high pretreatment weight loss; LWL, low weight loss during treatment; HWL, high weight loss during treatment; UW, underweight; NW, normal weight; OW, overweight; BMI, body mass index; HR, hazard ratio; CI, confidence interval.

## References

[1] Herszényi L., Tulassay Z. (2010). Epidemiology of gastrointestinal and liver tumors. *European Review for Medical and Pharmacological Sciences*.

[2] Cheng Y., Wang N., Wang K. (2013). Prognostic value of body mass index for patients undergoing esophagectomy for esophageal squamous cell carcinoma. *Japanese Journal of Clinical Oncology*.

[3] Di Fiore F., Lecleire S., Rigal O. (2006). Predictive factors of survival in patients treated with definitive chemoradiotherapy for squamous cell esophageal carcinoma. *World Journal of Gastroenterology*.

[4] Hayashi Y., Correa A. M., Hofstetter W. L. (2010). The influence of high body mass index on the prognosis of patients with esophageal cancer after surgery as primary therapy. *Cancer*.

[5] Jemal A., Center M. M., DeSantis C., Ward E. M. (2010). Global patterns of cancer incidence and mortality rates and trends. *Cancer Epidemiology, Biomarkers & Prevention*.

[6] Sun L., Zhang H., Wu K. (2014). Esophageal cancer: current options for therapeutic management. *Gastrointestinal Tumors*.

[7] Yildirim B. A., Özdemir Y., Colakoglu T., Topkan E. (2016). Impact of presence and degree of pretreatment weight loss in locally-advanced pancreatic cancer patients treated with definitive concurrent chemoradiotherapy. *Pancreatology*.

[8] Ghadjar P., Hayoz S., Zimmermann F. (2015). Impact of weight loss on survival after chemoradiation for locally advanced head and neck Cancer: Secondary results of a randomized phase III trial (SAKK 10/94). *Journal of Radiation Oncology*.

[9] Deans D. A. C., Tan B. H., Wigmore S. J. (2009). The influence of systemic inflammation, dietary intake and stage of disease on rate of weight loss in patients with gastro-oesophageal cancer. *British Journal of Cancer*.

[10] Van Der Schaaf M. K., Tilanus H. W., Van Lanschot J. J. B. (2014). The influence of preoperative weight loss on the postoperative course after esophageal cancer resection. *The Journal of Thoracic and Cardiovascular Surgery*.

[11] Hill A., Kiss N., Hodgson B., Crowe T. C., Walsh A. D. (2011). Associations between nutritional status, weight loss, radiotherapy treatment toxicity and treatment outcomes in gastrointestinal cancer patients. *Clinical Nutrition*.

[12] Yoon H. H., Lewis M. A., Shi Q. (2011). Prognostic impact of body mass index stratified by smoking status in patients with esophageal adenocarcinoma. *Journal of Clinical Oncology*.

[13] Blom R. L. G. M., Lagarde S. M., Klinkenbijl J. H. G., Busch O. R. C., Van Berge Henegouwen M. I. (2012). A high body mass index in esophageal cancer patients does not influence postoperative outcome or long-term survival. *Annals of Surgical Oncology*.

[14] Duan X.-F., Tang P., Shang X.-B., Jiang H.-J., Zhao Q., Yu Z.-T. (2017). High body mass index worsens survival in patients with esophageal squamous cell carcinoma after esophagectomy. *Digestive Surgery*.

[15] Miao L., Chen H., Xiang J., Zhang Y. (2015). A high body mass index in esophageal cancer patients is not associated with adverse outcomes following esophagectomy. *Journal of Cancer Research and Clinical Oncology*.

[16] Cincibuch J., Neoral C., Aujesky R. (2010). Prognostic factors in patients with esophageal carcinoma treated with chemoradiation: single center experience. *Hepatogastroenterology*.

[17] D’Journo X. B., Ouattara M., Loundou A. (2012). Prognostic impact of weight loss in 1-year survivors after transthoracic esophagectomy for cancer. *Diseases of the Esophagus*.

[18] Skipworth J., Foster J., Raptis D., Hughes F. (2009). The effect of preoperative weight loss and body mass index on postoperative outcome in patients with esophagogastric carcinoma. *Diseases of the Esophagus*.

[19] WHO Expert Consultation (2004). Appropriate body-mass index for Asian populations and its implications for policy and intervention strategies. *The Lancet*.

[20] Shen L.-J., Chen C., Li B.-F., Gao J., Xia Y.-F. (2013). High weight loss during radiation treatment changes the prognosis in under-/normal weight nasopharyngeal carcinoma patients for the worse: a retrospective analysis of 2433 cases. *PLoS ONE*.

[21] Edge S., Byrd D., Compton C., Fritz A., Greene F., Trotti A. *AJCC Cancer Staging Manual*.

[22] Rice T. W., Blackstone E. H., Rusch V. W. (2010). 7th edition of the AJCC cancer staging manual: esophagus and esophagogastric junction. *Annals of Surgical Oncology*.

[23] Beaver M. E. S., Matheny K. E., Roberts D. B., Myers J. N. (2001). Predictors of weight loss during radiation therapy. *Otolaryngology—Head and Neck Surgery*.

[24] Jiang N., Zhao J.-Z., Chen X.-C., Li L.-Y., Zhang L.-J., Zhao Y. (2014). Clinical determinants of weight loss in patients with esophageal carcinoma during radiotherapy: a prospective longitudinal view. *Asian Pacific Journal of Cancer Prevention*.

[25] Lee W. K., Hong S. K., Lee S. (2015). Prognostic value of body mass index according to histologic subtype in nonmetastatic renal cell carcinoma: a large cohort analysis. *Clinical Genitourinary Cancer*.

[26] Ren C., Cai X. Y., Qiu M. Z. (2015). mpact of body mass index on survival of esophageal squamous carcinoma patients in southern China. *Journal of Thoracic Disease*.

[27] Cvetanovic A., Popovic L., Filipovic S. (2015). Young age and pathological features predict breast cancer outcome—report from a dual institution experience in Serbia. *Journal of B.U.ON*.

[28] Abdollahi M., Hajizadeh E., Baghestani A. R., Haghighat S. (2016). Determination of a change point in the age at diagnosis of breast cancer using a survival model. *Asian Pacific Journal of Cancer Prevention: APJCP*.

